# Reply to Comment on “Binding Affinity Determines Substrate Specificity and Enables Discovery of substrates for N-Myristoyltransferases”

**DOI:** 10.1021/acscatal.2c01818

**Published:** 2022-07-08

**Authors:** Dan Su, Tatsiana Kosciuk, Hening Lin

**Affiliations:** † Department of Chemistry and Chemical Biology, Cornell University, Ithaca, New York 14853, United States; ‡ Howard Hughes Medical Institute; Department of Chemistry and Chemical Biology, Cornell University, Ithaca, New York 14853, United States

## Abstract

In our previously published article, an intriguing enzymology observation with the N-myristoyltransferases (NMT1 and NMT2) led us to conclude that binding affinity is important for determining in vivo substrate specificity and this can explain the vast literature that reports the coimmunoprecipitation of protein-modifying enzymes and their substrates. This understanding also provides a facile method to identify substrate proteins for such enzymes, which we demonstrated by identifying three substrate proteins using existing interactome data for NMT1 and NMT2. Dr. Meinnel recently commented on our finding, and we hope this Reply helps to clarify some of the important points we aimed to make in the original article.

We thank Dr. Thierry Meinnel for the interesting discussion on our published article entitled “Binding Affinity Determines Substrate Specificity and Enables Discovery of substrates for N-Myristoyltransferases”.^1^ It seems that Dr. Meinnel and we agree on the data but have different opinions on interpreting the data. In fact, several similar points were raised by the reviewers of our published paper, and we addressed the reviewers during the peer review process. It is important to emphasize that our article^1^ comes from a different perspective, which we will explain below in more detail.

Is *k*_cat_*/K*_m_ an important parameter? We agree with Dr. Meinnel that it certainly is despite the emphasis on *K*_d_ in our article. It is probably the most important parameter for developing biocatalysts for manufacturing purposes. Even for understanding the biological functions of enzymes at the cellular and organismal levels, *k*_cat_/*K*_m_ can still be useful and should continue to be measured. In many cases, *K*_m_ is likely a good estimate of *K*_d_.

The rationale for emphasizing *K*_d_ comes more from the perspective of the complex in vivo situation where multiple substrates compete for the same enzyme. The NMT case with acetyl-CoA versus myristoyl-CoA as substrates provides an intriguing example where *K*_m_ and *K*_d_ differ dramatically, highlighting the importance of *K*_d_ for in vivo specificity determination.

Why do we emphasize the importance of *K*_d_ in the paper? The main reason is a practical one. Currently, it is easy to identify the binding proteins for enzymes that control protein post-translational modifications (PTM), thanks to the advance of affinity-purification mass spectrometry (AP-MS). Based on the understanding that a good substrate should bind the enzyme well, we can predict that many of the interacting proteins could be substrates of the enzyme. Thus, we can identify substrate proteins for a PTM enzyme by identifying its interacting proteins. In the second half of the paper, we used the publicly available NMT interactome data to illustrate that this is indeed feasible.^1^

This point may be of limited utility for identifying NMT substrates as the work by several groups, including Dr. Meinnel’s, have provided a rather comprehensive picture of what proteins are myristoylated by NMT. For NMT, there are clear and well-understood N-terminal sequence preferences and the use of alkyne-tagged myristic acid analogues to help identify substrate proteins. However, for other PTM enzymes, such as kinases, phosphatases, methyltransferase, demethylases, acetyltransferases, and deacetylases, with a lack of well-defined sequence preference or effective affinity purification reagents for the PTMs, substrate identification can be very difficult. This is why many chemical biology laboratories have spent much effort developing different technologies, such as the bump-and-hole method for identifying substrates for kinases,^2^ methyltransferases,^3^ and ADP-ribosyltransferases.^4^ Such technologies require specific engineering efforts for each enzyme of interest and thus require substantial efforts. Now with the understanding that a good substrate should also bind the enzyme well, interactome data, which can be readily obtained with modern proteomics technologies, can significantly speed up the discovery of new substrate proteins.

Furthermore, emphasizing *K*_d_ can explain the vast literature that reports the coimmunoprecipitation of PTM enzymes and their substrates, which we mentioned in the article.^1^ For example, our lab works on a class of deacylases called sirtuins. We noticed that for almost all identified sirtuins substrates, their coimmunoprecipitation with the sirtuin enzyme was reported.^5−8^ This is also true for other PTM enzyme–substrate pairs.^9−11^ Thus, we believe this is a rather general phenomenon in biology. This cannot be explained by emphasizing *k*_cat_*/K*_m_ but can be explained by emphasizing *K*_d_.

Below are our responses to the specific important points made by Dr. Meinnel.

- Drastic difference in *K*_d_ values for acyl CoAs of NMTs does not exclusively account for the substrate selectivity. Other mechanisms, such as ACBD6, Molecular crowding, pH, or salt concentrations, may account for in vivo substrate specificity.

While we agree that other mechanisms may also account for the in vivo substrate specificity, these mechanisms are yet to be experimentally tested. The ACBD6 speculation is interesting as there is a report suggesting that ACBD6 may promote myristoylation over palmitoylation.^12^ Palmitoyl-CoA can also be used as a substrate by NMT, and in cells, palmitoyl-CoA is present at higher concentrations than myristoyl-CoA. ACBD6, which binds longer chain fatty acyl-CoA better than shorter ones, interacts with NMT and can sequester and shelter NMT from palmitoyl-CoA, thus promoting myristoylation in cells.^12^ However, this claim has not been widely validated by other laboratories. If this is true, it would actually help to promote acetylation by NMT if we use the same argument for how ACBD6 promotes myristoylation over palmitoylation, because ACBD6 should binds to myristoyl-CoA better than acetyl-CoA and thus shelter NMT from myristoyl-CoA. Apparently, this is not what happens in cells.

When making this point, Dr. Meinnel assumed that the two acyl-CoAs are present in eukaryotic cytosols at concentrations similar to their binding affinities. If that is the case, then indeed the binding affinity alone would not be able to explain the myristoylation specificity and other mechanisms would need to be involved. It is surprisingly difficult to estimate the free acyl-CoA concentrations in cells because long-chain fatty acyl-CoA can bind to many proteins and membranes and thus effectively buffered.^13^ Thus, it is not clear whether the assumption is correct. In enzymology, we typically assume that the physiological concentration of a substrate is around the *K*_m_ value (not the *K*_d_ value), but even that is not true in many cases. For example, many protein kinases have *K*_m_ values for ATP (μM) that are much lower than the physiological concentration of ATP (mM).^14^ In the NMT case, even if the cellular myristoyl-CoA concentration is 200 nM, which falls within the estimated values,^13^ then NMT would be mostly occupied by myristoyl-CoA and catalyze myristoylation selectively.

- *k*_cat_/*K*_peptide_ accounts for the substrate specificity for acyl-CoAs. The reviewers for our paper also raised this point during the peer review process and we addressed this. The *k*_cat_*/K*_peptide_ for acetylation is about 23-fold lower than that for myrisotylation, which could potentially explain the in vivo substrate specificity.^1^ This 23-fold difference is mainly because the *K*_peptide_ for acetylation is 40 μM, while that for myristoylation is 5 μM (∼8-fold difference). However, in Figure 2 of the paper, we added both acetyl-CoA and myristoyl-CoA in the reaction, and essentially only the myristoylation product formed.^1^ In this reaction, we used 200 μM of peptide (5 times of *K*_peptide_), and thus, the reaction rate should be mainly determined by *k*_cat_. The *k*_cat_ difference for acetylation and myristoylation is only ∼3-fold, which cannot explain the fact that we saw mostly myristoylation product in the reaction with a saturating concentration of peptide. The fact that we still see mostly myristoylation product under this condition suggests that the binding competition between acetyl-CoA and myristoyl-CoA is the main reason for the preferential myristoylation reaction.
- “With all the arguments above, *k*_cat_/*K*_M_ measurements in vitro were and still are a relevant strategy for discovering or validating NMT substrates with significant sampling and discovery rate.” As explained in the beginning, we are not saying that *k*_cat_*/K*_m_ measurement is not important anymore. Under many circumstances, measuring *k*_cat_*/K*_m_ will provide useful information. We emphasize that measuring *k*_cat_*/K*_m_ does not provide a facile method to discover unknown substrate proteins for PTM enzymes (especially enzymes other than NMT), but binding affinity can, because current proteomic technologies allow routine interactome identification for any protein of interest.
- “The study does not demonstrate that the binding affinity underpins the discovery of NMT substrates. Only the strong binding affinity of the product to the enzyme explains the data”. We agree with Dr. Meinnel that for NMT, the myristoylated protein may bind better than the unmyristoylated substrate. This is because the binding affinity to NMT comes from two parts, the substrate protein and the myristoyl group. The presence of myristoyl group on the product will likely increase the binding affinity to NMT because of hydrophobic interactions with the acyl pocket. However, our conclusion that a substrate with higher binding affinity would be a more preferred substrate in vivo still stands for the following reasoning: if myristoyl-protein A binds to NMT better than myristoyl-protein B does, in most cases we can assume that protein A also binds to NMT better compared to protein B, and thus, Protein A is a preferred substrate. In other words, for NMT (and likely also for other PTM enzymes), relative protein product binding affinity in most cases also reflects relative protein substrate binding affinity.
- “The authors missed that all these “new” substrates had already been identified in previous studies. I acknowledge that none of the missing three entries were in the 121 experimentally validated myristoylated human proteins available in UniProt (Table S1). This further strengthens the urgent need to improve data annotation in protein data resources.” We are grateful to Dr. Meinnel for pointing out that the three substrates we identified and validated came up in previous omics studies. We apologize for the oversight. We missed them because they were listed under different names (and even under different UniProt # for one of them). The articles that Professor Meinnel mentioned also found these proteins could be potential substrates using high-throughput approaches for myristoylome interrogation. However, new hits from such omics studies should only be considered as putative substrates of NMT before further validation. For instance, an in vitro reaction using a peptide substrate may not be reflective of what occurs in a biological setting. Our study supports this notion by demonstrating that NMT can efficiently acetylate the N-terminal peptide of ARF6 in vitro, but this does not happen in cells, at least under conditions we tested. Furthermore, high-throughput substrate identification approaches are known to have artifacts that cannot be ruled out without validation studies. Therefore, it is fair to say that using the available interactome data, we identified and validated three substrates of NMT that were previously suggested to be NMT substrates by other omics data. It is indeed true that these entries remain absent from UniProt, pointing to limitations in protein data annotation. We are therefore appreciative of Dr. Meinnel’s recommendation toward overcoming this challenge by compiling all available myristoylation data.
- “While Su et al. report two very interesting observations related to NMT, these are unrelated to each other.”

A similar comment was raised during the peer review process, and we addressed this. We gladly clarify the connection between the two observations here. We aimed to convey that just like the differences in the binding affinity of various Acyl-CoA types dictate which type is a preferred Acyl-CoA substrate, the differences in binding affinities of protein substrates might predict preferred protein substrates.

From the acyl-CoA studies with NMT, we learned that binding affinity could be a better predictor than *k*_cat_*/K*_m_ for determining substrate specificity in vivo. We believe this might be generally applicable to in vivo substrate specificity of many PTM enzymes. An inference from this is that a more preferred substrate protein will bind to its enzyme more tightly. Thus, by searching for proteins that bind to a PTM enzyme, we can identify some of its preferred substrates in vivo. This notion is supported by the vast literature reporting that PTM enzymes can coimmunoprecipitate with their substrate proteins. To further explore this possibility, we used the known interactome data of NMT1 to identify previously unknown substrate proteins. This data mining exercise revealed many interactors that are known NMT1 substrates, supporting our hypothesis. Furthermore, we were able to identify and validate three previously unknown/unvalidated proteins modified by NMT, highlighting the utility of interactome approaches in enzyme substrate searches.

In other words, our acyl-CoA studies with NMT illustrate that binding affinity is an important parameter for determining substrate specificity in vivo and can be a better predictor of preferred substrates. Our NMT substrate identification effort further confirmed this view and demonstrated its practical utility that might be of great importance to the PTM enzyme fields, in which identifying the substrate proteins remain an important and difficult task.

We again wish to thank Dr. Thierry Meinnel for the interesting discussion, which clarifies and enhances some of the important points we made in the original article. We believe this dialogue may help readers understand our findings.
